# A tailored phase I-specific patient-reported outcome (PRO) survey to capture the patient experience of symptomatic adverse events

**DOI:** 10.1038/s41416-023-02307-w

**Published:** 2023-07-07

**Authors:** Helena J. Janse van Rensburg, Zhihui Liu, Geoffrey A. Watson, Zachary W. Veitch, Daniel Shepshelovich, Anna Spreafico, Albiruni R. Abdul Razak, Philippe L. Bedard, Lillian L. Siu, Lori Minasian, Aaron R. Hansen

**Affiliations:** 1 Division of Medical Oncology and Hematology, Princess Margaret Cancer Centre, University Health Network, University of Toronto, Toronto, ON Canada; 2 Department of Biostatistics, Princess Margaret Cancer Centre, University Health Network, University of Toronto, Toronto, ON Canada; 3 Medicine D, Tel-Aviv Medical Center and the Sackler School of Medicine, Tel Aviv, Israel; 4 National Cancer Institute, National Institutes of Health, Bethesda, MD USA; 5 Faculty of Medicine, University of Queensland, Brisbane, QLD Australia

**Keywords:** Drug development, Phase I trials, Outcomes research

## Abstract

**Background:**

Patient perspectives are fundamental to defining tolerability of investigational anti-neoplastic therapies in clinical trials. Phase I trials present a unique challenge in designing tools for efficiently collecting patient-reported outcomes (PROs) given the difficulty of anticipating adverse events of relevance. However, phase I trials also offer an opportunity for investigators to optimize drug dosing based on tolerability for future larger-scale trials and in eventual clinical practice. Existing tools for comprehensively capturing PROs are generally cumbersome and are not routinely used in phase I trials.

**Methods:**

Here, we describe the creation of a tailored survey based on the National Cancer Institute’s PRO-CTCAE for collecting patients’ perspectives on symptomatic adverse events in phase I trials in oncology.

**Results:**

We describe our stepwise approach to condensing the original 78-symptom library into a modified 30 term core list of symptoms which can be efficiently applied. We further show that our tailored survey aligns with phase I trialists’ perspectives on symptoms of relevance.

**Conclusions:**

This tailored survey represents the first PRO tool developed specifically for assessing tolerability in the phase I oncology population. We provide recommendations for future work aimed at integrating this survey into clinical practice.

## Background

The primary objective of phase I trials in oncology is to test the safety and tolerability of investigational anti-cancer agents. Drug safety is interrogated through clinical and laboratory evaluation, allowing dose-limiting toxicities to be identified and a recommended phase II dose to be determined. Robust tools exist for characterizing safety of an experimental drug, such as the Common Terminology Criteria for Adverse Events (CTCAE) [1]. In contrast, agent tolerability has historically represented an endpoint that is ambiguously described by investigators, where the term is often misused in summarizing a favourable safety profile rather than encompassing patient perspectives on the adverse effects of an agent [2].

In recent years, there has been a growing appreciation for the role of patient-reported outcomes (PROs) in defining tolerability [3–6]. Indeed, organizations including the U.S. Food and Drug Administration now recommend the inclusion of such data within published trials [7–10]. However, standardized methods for assessing tolerability using PROs in phase I trials are lacking.

The PRO-CTCAE is a 124-item library developed by the National Cancer Institute (NCI) which probes the presence/absence, severity, interference on activities, frequency, and amount of 78 symptoms described in 80 “terms” [11]. Since its inception, the PRO-CTCAE has been well-validated and translated into more than 30 languages [12–15]. Interestingly, when using this tool, we and others have noted variability between provider- and patient-perspectives on adverse events (AEs), underscoring the importance of this type of assessment in describing tolerability [16–19].

Despite this, the integration of PRO tools into trial design has been hindered by their generally lengthy nature, which requires a considerable time investment by providers and patients [20]. Although we have shown that the full PRO-CTCAE can be feasibly administered in phase I trials, it is rarely used in this context [21–23]. In fact, a systematic review of phase I trials on *clinicaltrials.gov* revealed that only 2.3% included at least one PRO of any type [23]. In the few studies which did include PROs, no single standard tool was applied. Indeed, phase I trials present a unique challenge for designing such a tool as first-in-human studies with tremendous uncertainty around anticipated AEs. In order to capture valuable patient perspectives on tolerability in phase I trials in a standardized manner, a tailored survey which balances comprehensiveness with efficiency, prioritizing symptoms most affecting tolerability, will be required.

In the present study, we describe the creation of such a tailored survey. We use primary patient data to identify a core set of symptoms warranting inclusion and further align this with phase I trialists’ perspectives on symptoms most impacting tolerability. Finally, we offer directions for future work integrating this survey into practice.

## Methods

### Source data

The source data for the present study was obtained from our previous prospective, single-center, observational investigation of patient/physician agreement in AE reporting in phase I trials [19]. Adult patients were approached for participation in an outpatient, centralized phase I clinic at the Princess Margaret Cancer Centre in Toronto, Ontario from May 1, 2017, to January 1, 2019. Inclusion criteria were phase I trial enrolment, English fluency, and absence of clinically significant cognitive impairment. Further data related to patient characteristics were collected. Evaluation of response was performed by the study investigator of this trial, using RECIST v1.1 criteria.

The PRO-CTCAE (https://healthcaredelivery.cancer.gov/pro-ctcae/) was administered to patients electronically using tablet computers at three timepoints: before investigational therapy was initiated (baseline), mid-cycle 1, and mid-cycle 2. The full PRO-CTCAE including 124 survey items corresponding to 80 terms (78 symptomatic AEs with two terms each for depression and irregular menstruation) was used. For each term up to three survey items are included to characterize symptom attributes including the presence (“yes” or “no”), severity (“none”, “mild”, “moderate”, “severe”, or “very severe”), interference on usual or daily activities (“not at all”, “a little bit”, “somewhat”, “quite a bit”, or “very much”), frequency (“never”, “rarely”, “occasionally”, “frequently”, or “almost constantly”), or amount (“not at all”, “a little bit”, “somewhat”, “quite a bit”, or “very much”) of that symptom.

Patients were informed that their responses would not be known by the clinical team and the team was blinded to patients’ responses. Demographic and survey data were housed in a centralized database. Survey responses were adjusted for attrition in patients who discontinued participation before the mid-cycle 1/2 timepoints.

### Statistical tailoring

The pooled PRO-CTCAE survey responses from the three timepoints were used in subsequent analyses using R version 4.0.2 [24], R package *psych* [25], and Microsoft Excel platforms. Descriptive statistics were generated, and survey terms were subjected to iterative rounds of ranking and elimination as follows:

(i) *AE prevalence* as calculated by the number of responses with severity > “none”, interference > “not at all”, frequency > “never”, or amount > “not at all” (i.e., the presence of any attribute) divided by the total number of survey responses OR for terms with absence/presence questions only, the number of survey responses “yes” divided by the total number of survey responses. A threshold for elimination of 5% was chosen, based on commonly-used definitions of AE prevalence and literature describing patient interpretations of AE frequency [26].
(ii) *severity proportion score* as calculated by the number of responses with severity ≥ “moderate” divided by the number of responses with severity > “none”. In phase I trials, a maximum tolerated dose for an investigational agent is defined by the proportions of patients experiencing dose-limiting toxicities. A threshold of ≤20–33% is generally considered to be acceptable in this setting [27]. To parallel this, we selected a threshold of 25% for elimination (i.e., if >75% of patients experiencing a symptom described the severity of that symptom as “mild”, that term was eliminated). Terms meeting the threshold for inclusion in step (i) but that did not include a severity question were retained on this step and were allowed to move forward to step (iii).
(iii) *interference proportion score* as calculated by the number of responses with interference ≥ “somewhat” divided by the number of responses with interference > “not at all”. A threshold of 25% was used for elimination. Terms meeting the thresholds for inclusion in both steps (i) and (ii) but that did not include an interference question were retained on this step and were allowed to move forward to step (iv).
(iv) *frequency proportion score* as calculated by the number of responses with frequency ≥ “occasionally” divided by the number of responses with frequency > “never”. A threshold of 25% was used for elimination. Terms meeting the thresholds for inclusion in all of steps (i)–(iii) but that did not include a frequency question were retained on this step and were allowed to move forward to step (v).
(v) *amount proportion score* as calculated by the number of responses with amount ≥ “somewhat” divided by the number of responses with amount > “not at all”. A threshold of 25% was used for elimination. Terms meeting the thresholds for inclusion in all of steps (i)–(iv) but that did not include an amount question were retained on this step and were allowed to move forward to step (vi).
(vi) *percent change in domain reliability*. Within survey research, reliability (calculated as Cronbach’s alpha, (α)) describes whether an instrument measures a concept consistently [28]. The 80 PRO-CTCAE terms are grouped into organ system domains. Terms which can be removed with minimal effect on that domain’s reliability are likely low yield for inclusion on a tailored survey. The domain reliability for each domain was calculated (α_1_), and then was re-calculated with single terms independently excluded from each domain (α_2_). Change in reliability (Δα) for each term was calculated as α_2_ − α_1_. Percent change in domain reliability for each term was calculated Δα/α_1_ (expressed as a percent). One term with lowest percent change in domain reliability from each domain was removed. No terms were removed from domains with ≤2 remaining terms as this would have a substantial impact on reliability.

### Survey of phase I investigators

To collect trialists’ perspectives on the tailored list of symptoms, an electronic survey was created with standard methodology [29]. 24 trialists were identified as having experience in the design and implementation of phase I trials and were invited for participation through direct communication. Nine investigators agreed to participate. Survey participants answered questions related to their experience in phase I trials and were asked to rate the impact on tolerability of terms from the PRO-CTCAE. To further explore survey responses, individual videoconference interviews were performed and transcribed. Participants were asked about AEs they described as being impactful and not impactful on tolerability in phase I trials, as well as about their experiences and opinions on the integration of PRO tools into phase I trial design. The study protocol received institutional ethics approval. Informed consent was obtained, and responses were housed in a centralized database.

Survey and interview responses were subjected to descriptive statistics. For term elimination, physician-reported impact proportion scores were calculated as the number of responses with impact on tolerability ≥ “moderate” divided by the total number of responses. A threshold of 25% was used for elimination.

## Results

### Source data

To generate a tailored PRO-CTCAE survey informed by patient perspectives, we used raw data from our previous investigation of patient/physician agreement in AE reporting in phase I trials [19]. In this tumor type- and investigational therapy-agnostic study, patients in phase I trials completed the full PRO-CTCAE at baseline, mid-cycle 1, and mid-cycle 2, and survey responses were compared against physician assessments.

Data from 528 surveys (219 baseline, 191 mid-cycle 1, 118 mid-cycle 2) representing responses from 219 patients were included in our analysis (Table 1). The median age of participants was 60, with 50.7% male and 49.3% female patients. The most common oncologic sites were gastrointestinal, head and neck, and breast. Most investigational agents were administered as combination therapies, and many were immunotherapy-based. Targeted therapies were less common. Finally, the overall response rate in our population was 9.6%.

**Table 1:** Patient, treatment, and response characteristics (*n* = 219).

Characteristic	No. (%)
Median age at enrollment [range]	60 [18–82]
Gender	
Male	111 (50.7)
Female	108 (49.3)
ECOG	
0	47 (21.5)
1	172 (78.5)
Education level	
Elementary school	4 (1.8)
High school	67 (30.6)
Postgraduate (Non-university)	52 (23.7)
University	96 (43.8)
English as first language	
Yes	194 (88.6)
No	25 (11.4)
Oncologic site	
Gastrointestinal	63 (28.8)
Head and neck	30 (13.7)
Breast	23 (10.5)
Genitourinary	22 (10.0)
Gynecological	21 (9.6)
Melanoma	18 (8.2)
Lung	17 (7.8)
Sarcoma	17 (7.8)
Other	8 (3.6)
Monotherapy or combination	
Monotherapy	79 (36.1)
Combination	140 (63.9)
Treatment type	
Immunotherapy-based	144 (65.8)
Targeted-based	53 (24.2)
Immuno-targeted combination	22 (10.0)
Best response^a^	
CR	3 (1.4)
PR	18 (8.2)
SD	79 (36.1)
PD	117 (53.4)
NE	2 (0.9)

^a^Response Evaluation Criteria in Solid Tumours (RECIST) v1.1. *ECOG* Eastern Cooperative Oncology Group, *NE* not evaluable, overall response rate (complete response + partial response) = 9.6%.

### Survey tailoring

**Fig. 1:**
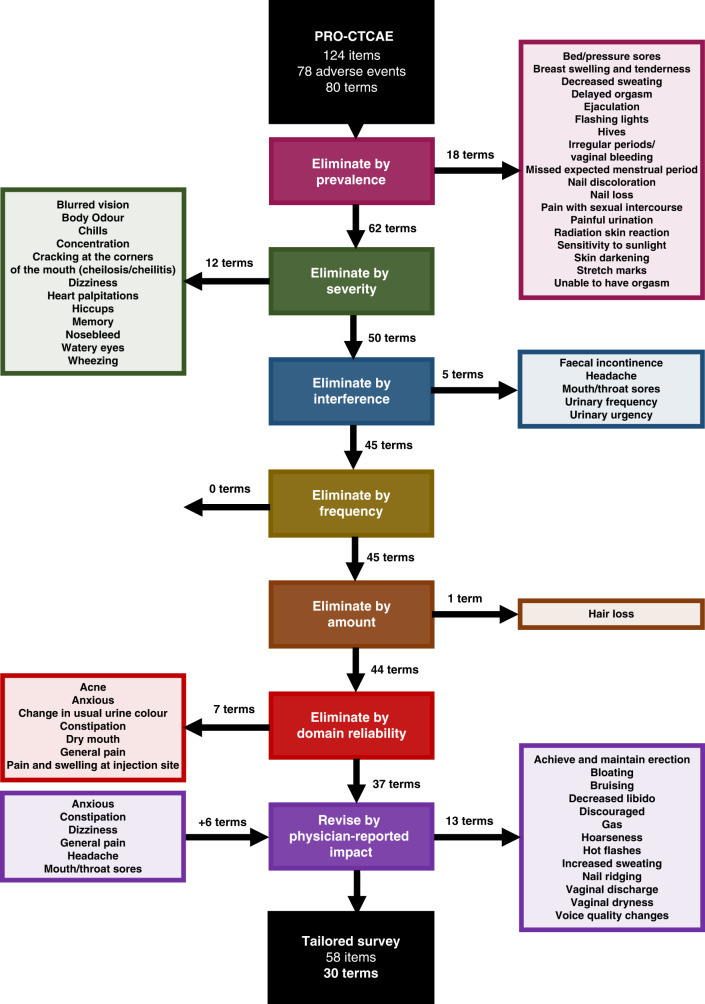
Approach to creation of a tailored PRO-CTCAE survey for cancer patients participating in phase I clinical trials. In brief, the full length, 124-item PRO-CTCAE representing 78 adverse events described in 80 terms was subjected to multiple iterations of tailoring. First, low prevalence terms experienced by <5% of patients in the study population were eliminated. In subsequent steps, terms with low severity, interference, frequency, or amount proportion scores (<25%) amongst the study population were eliminated. Terms with a small effect on overall symptom domain reliability when removed were next excluded. Finally, terms with low physician-reported impact on tolerability were removed and 6 terms repeatedly mentioned by trialists as being relevant to include were re-introduced. Eliminated or re-introduced terms are listed beside the corresponding step during which they were removed or added, respectively.

In refining the PRO-CTCAE (Fig. 1), we reasoned that a tailored tool should capture AEs most likely to occur in the phase I population and established a threshold prevalence below which terms would be removed. Using a threshold of 5%, 18 terms were eliminated. Multiple cutaneous symptoms were amongst the most uncommon terms reported (hives (4.7%), sensitivity to sunlight (4%), radiation skin reaction (3.6%), nail discoloration (2.8%), skin darkening (2.5%), stretch marks (2.5%), nail loss (1%), bed pressure sores (0.8%)). Several genitourinary (painful urination (3.4%), irregular periods/vaginal bleeding (1.3%), missed expected menstrual period (1.3%)) and sexual symptoms (ejaculation (4.2%), unable to have orgasm (3%), pain with sexual intercourse (3%), delayed orgasm (2.6%)) were also eliminated by this criterion. Two miscellaneous terms (breast swelling and tenderness (4.4%), decreased sweating (2.1%)) and one visual/perceptual term (flashing lights (2.6%)) were also eliminated for low prevalence in our study population (Supplementary Fig. 1).

Having removed uncommon terms, we next decided that our survey should prioritize AEs with highest severity as these would intuitively be most likely to affect tolerability. To quantify the overall severity of a particular symptom within the study population, we created a severity proportion score for each survey term. This value is calculated by determining the proportion of patients who reported symptom severity ≥ “moderate” for a particular symptom amongst all patients who experienced that symptom. For example, a severity proportion score of 39.4% for fatigue can be interpreted as 39.4% of all patients who reported having fatigue described this as being at least “moderate” in severity. Moreover, the severity proportion score of 39.4% for fatigue further corresponds to 60.6% of patients experiencing fatigue that they described as “mild” in severity. Using a threshold severity proportion score of 25%, 12 survey terms were removed (Supplementary Fig. 2). These included blurred vision (8.2%), body odor (6.4%), chills (23.2%), concentration (13.4%), cracking at the corners of the mouth (cheilosis/cheilitis; 23.3%), dizziness (16.7%), heart palpitations (18.5%), hiccups (17.9%), memory (11.7%), nosebleed (10.5%), watery eyes (10.3%), and wheezing (19.2%). Of note, three survey terms that were eliminated for prevalence <5% in the study population had severity proportion scores above the ≥25% threshold signaling that these adverse events may still be of value to inquire about outside of a tailored tool. These were pain with sexual intercourse (43.8%), breast swelling and tenderness (39.1%), and radiation skin reaction (31.6%). In reviewing the primary survey data for each of these terms, most survey respondents with ≥ “moderate” symptom severity in fact rated their symptom as “moderate” with “severe” or “very severe” symptom severity responses being infrequent for each of these terms (proportion of patients who reported ≥ “severe” amongst all patients who experienced that symptom: pain with sexual intercourse (12.5%), breast swelling and tenderness (8.7%), and radiation skin reaction (10.5%)).

We further incorporated patient descriptions of AE interference, frequency, and amount into our survey tailoring. We created interference, frequency, and amount proportion scores to quantify these attributes and applied a threshold of <25% for term revision.

Five survey terms were eliminated for low interference (Supplementary Fig. 3). These included two terms from the genitourinary organ system domain (urinary frequency (23.5%), urinary urgency (16.2%)), as well as terms from the oral (mouth/throat sores (23.3%)), gastrointestinal (fecal incontinence (21.4%)), and pain (headache (21%)) domains. Other terms with low interference proportion scores had already been eliminated by low prevalence or severity proportion scores, although having a low interference proportion score further justifies the removal of these terms. These included concentration (17.8%), dizziness (16.7%), memory (15.2%), blurred vision (7.5%), and watery eyes (2.3%). There were no survey terms that had been eliminated in previous tailoring steps that had an interference proportion score ≥25%.

Only two survey terms had frequency proportion scores that fell below the 25% threshold—urinary urgency (23.7%) and nosebleed (16.7%) (Supplementary Fig. 4). Interestingly, both survey terms had already been removed in previous analysis steps, although their having low frequency proportion scores further justifies their exclusion from the tailored survey. Several terms that had been eliminated in previous steps were found to have a frequency proportion score ≥25%. These included urinary frequency (43.5%), headache (37.4%), ejaculation (36.4%), chills (31.1%), hiccups (29.4%), heart palpitations (29.3%), and fecal incontinence (25%). In reviewing the primary survey data for each of these terms, most survey respondents with ≥ “occasionally” symptom frequency in fact rated their symptom as “occasionally” with “frequently” or “almost constantly” symptom frequency responses being uncommon for each of these terms (proportion of patients who reported ≥ “frequently” amongst all patients who experienced that symptom: urinary frequency (15.3%), headache (5.2%), chills (4.2%), hiccups (7.4%), heart palpitations (0%), and fecal incontinence (3.1%)). Despite being described as relatively frequent, each of these terms did not meet the severity or interference proportion score thresholds in prior steps to warrant inclusion on the tailored survey. An exception to this was ejaculation, which a larger proportion of patients described as occurring ≥ “frequently” amongst all patients experiencing this symptom (22.7%), but which had been removed due to low prevalence within the study population (4.2%) and lacked severity or interference attributes questions for further characterization.

Only two survey terms on the full length PRO-CTCAE include an amount question: vaginal discharge and hair loss (Supplementary Fig. 5). For vaginal discharge, 68.6% of patients experiencing this symptom reported it being at least “somewhat” in amount. In contrast, 16.4% of patients experiencing hair loss described this as being at least “somewhat” in amount and, therefore, the hair loss survey term was removed from our tailored survey. Of note, 9.0% of patients experiencing hair loss described their hair loss as being at least “quite a bit”, identifying this symptomatic adverse event to be one of concern that might be included on more comprehensive tools.

Finally, we used domain reliability to identify terms within each organ system domain which were redundant or low yield for inclusion. In this way, we removed seven further terms from the tailored survey (acne, anxious, change in usual urine color, constipation, dry mouth, general pain, pain and swelling at injection site) (Supplementary Fig. 6).

### Refining the tailored survey with perspectives from investigators

Having used patient data to generate a list of 37 terms of interest, we next explored whether the component AEs aligned with phase I trialists’ perspectives on symptoms relevant to tolerability in this population. We created a survey in which physician participants were asked to rate the impact on tolerability of each of the 37 terms and to identify other symptoms impacting tolerability. We further conducted interviews with each participant to explore their perspectives on PRO tools in phase I trials and to obtain feedback on the tailored list and our approach to its creation.

Nine participants completed the survey and interview. These included trialists from tertiary centers in North America, Europe, and Australia, who had a cumulative 172 years of experience in treating cancer patients, 133 years of experience in working in phase I trials, and who had collectively treated at least 770 patients in this context (Table 2). Most had never used PRO tools in their phase I trial design. Commonly described barriers included a lack of a standardized tool, the length of existing comprehensive tools with competing demands on time, and heterogeneity of the patient population and therapeutics. Despite these challenges, several interviewees highlighted the anticipated value of collecting PROs, particularly where standard methods for evaluating AEs might under-estimate their true impact on tolerability.

**Table 2:** Phase I trialists characteristics (*n* = 9).

Characteristic	No. (%)
Experience working with cancer patients, years	
Median [range]	20 [6–30]
Cumulative	172
Experience working in phase I trials, years	
Median [range]	15 [3–20]
Cumulative	133
Cumulative number of phase I patients treated in phase I trials	>770
Experience using PRO tools in phase I trials	
Have never used	5 (55.6%)
Have used 1-2 times	4 (44.4%)
Routinely use	0 (0%)

Survey respondents were also asked about potential methods for tailoring symptom lists. All survey respondents agreed that a prevalence threshold of approximately 10% would be acceptable for capturing most AEs of interest. When asked about an appropriate survey length for feasible administration in a phase I trial setting, the average number provided was 26 items (range 10–50).

We further created a physician-reported impact proportion score to quantify the overall impact of a particular symptom on tolerability and used a threshold of 25% to eliminate terms. By this criterion, 13 terms were removed from the tailored survey (achieve and maintain erection (11.1%), bloating (0%), bruising (11.1%), decreased libido (11.1%), discouraged (11.1%), gas (0%), hoarseness (22.2%), hot flashes (22.2%), increased sweating (11.1%), nail ridging (0%), vaginal discharge (22.2%), vaginal dryness (11.1%), voice quality changes (22.2%)) (Supplementary Fig. 7). We were also able to identify symptoms omitted from our list which have a moderate or major impact on tolerability in the investigators’ experiences. The terms anxious, constipation, dizziness, general pain, headache, and mouth/throat sores were reintroduced into the tailored list based on their being repeatedly mentioned by the investigators.

**Fig. 2:**
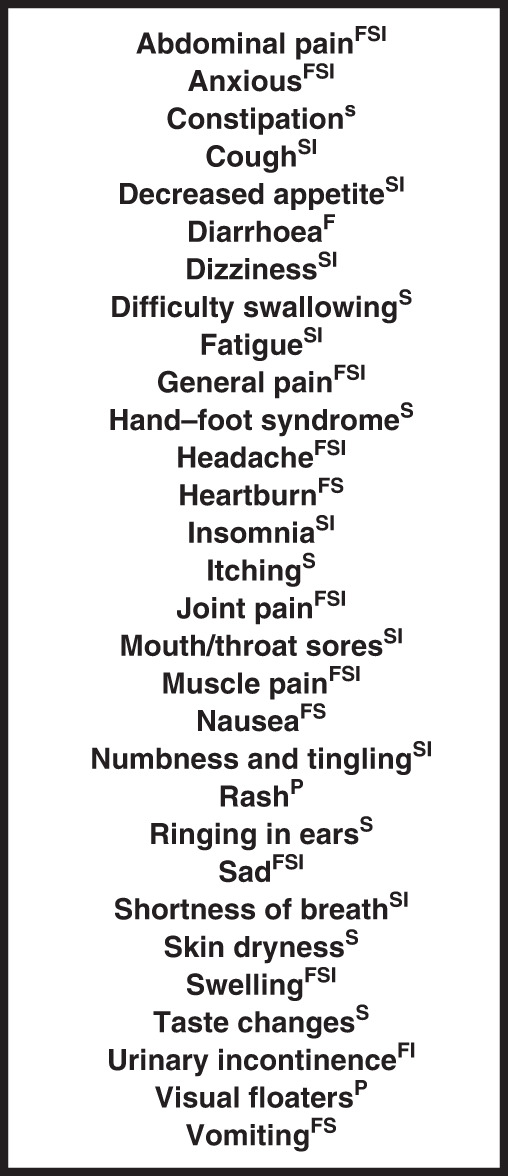
List of survey terms in a tailored PRO-CTCAE survey for phase I trial cancer patients. 30 terms corresponding to 58 potential survey items are included in the tailored survey. Attribute questions to be included for each term are denoted in superscript (F frequency, I interference, P presence, S severity). Frequency, interference, and severity questions are scored from 0–4 as per the full length PRO-CTCAE whereas presence/absence questions are scored from 0–1.

The resultant final tailored PRO-CTCAE for phase I trials (Fig. 2) is comprised of 30 symptomatic AE terms from 11 organ system domains and corresponds to a maximum survey length of 58 questions if all AEs are present and conditional branching is not used for administration.

## Discussion

In the present study, we describe the development of a tailored survey for capturing PROs amongst phase I trial patients receiving experimental anti-cancer agents. Our core list of symptoms was condensed from the NCI’s PRO-CTCAE symptom library and is informed by patient data and phase I trialists’ experiences. To the best of our knowledge, this survey represents the first PRO tool developed specifically for assessing tolerability in the phase I oncology population.

Phase I trials represent a valuable opportunity for the recognition of patient-reported symptomatic AEs. When compared to investigator assessments, PROs provide a more accurate definition of how therapy is received from the patient perspective where drug-related AEs are contextualized amongst various factors influencing tolerability (e.g., concurrent symptoms from malignancy or comorbidity). Favorable PRO data may be useful in justifying drug regulatory approval [30, 31]. Finally, the use of PROs in defining dose-limiting toxicities may inform recommended phase II doses with improved tolerability which could conceivably affect time on therapy and survival outcomes.

In generating our core list of symptoms, we reasoned that AEs should have adequate prevalence to warrant inclusion. We selected a threshold of 5% for term elimination which was informed by commonly-used definitions of AE frequency. This threshold is also reflective of the small sample sizes generally used in phase I trials (i.e., one patient experiencing an AE amongst a sample size of 20 equates to 5% prevalence), and is more inclusive than a previously proposed 10% threshold for term selection in early-phase oncology trials [32]. The interviewed trialists generally agreed with our approach, and repeatedly reinforced the importance of not missing severe but uncommon events. Reassuringly, only three terms eliminated based on prevalence had severity proportion score ≥25% and in reviewing the primary data, most of these patients did not have “severe” or “very severe” survey responses. Furthermore, none of the terms eliminated by prevalence exceeded the interference proportion score threshold, which would allow us to conclude that we did not remove rare severe/highly-interfering AE terms.

We subsequently proceeded through rounds of term ranking and elimination by AE severity, interference, frequency, and amount. In each of these steps, proportion scores were used to quantify the relative magnitude of symptom burden and a threshold of 25% was used to eliminate terms. In oncology trials, a threshold of ≤20–33% of patients experiencing a particular dose-limiting toxicity is generally considered to be an acceptable threshold for selecting a maximum-tolerated dose. Our 25% threshold reflects this and results in the capture of lower grade toxicities that do not meet the usual definitions for dose-limiting toxicities. For example, moderately severe (CTCAE grade 2) abdominal pain would be counted towards the numerator of our severity proportion score calculation but would not otherwise meet the definition for a dose-limiting toxicity unless prolonged or interfering.

In our final tailoring step, we sought the perspectives of phase I trialists. Recognizing that the PRO tool would ultimately be applied by trialists, we felt it should be refined in this manner. In considering how to utilize the physician data, we were cautious about having physicians’ perspectives outweigh the patient data informing term selection to this point. However, in reviewing the terms removed in this step, we noted that multiple symptoms (e.g., bruising, gas, nail ridging) were not associated with any severity/interference/frequency/amount attributes and had only been included in the 37-term list due to having prevalence >5%. Other terms (e.g., bloating, hoarseness, vaginal dryness) only had one associated attribute question for which the proportion score exceeded 25%.

The resultant 30-term list aligns with the sampled investigators’ expectations for a tailored tool, and is of comparable length to previously created study-specific surveys [13, 33]. Our symptom list includes 11/12 symptomatic adverse events from a recommended core set of symptoms to include in adult oncology trials, with cognitive problems (i.e., memory and concentration) being the only terms not meeting thresholds for inclusion in our study [34]. While a tailored tool cannot exhaustively capture all symptoms impacting tolerability, tools such as the full PRO-CTCAE already exist for this purpose and could be applied alongside our tailored survey at critical timepoints in a trial to balance completeness with efficiency. If there is a reasonable suspicion that an experimental therapy will have an AE of interest that is not covered by our tailored list, or that certain disease-related symptoms might be present at baseline, then additional specific terms could be added to our questionnaire, in keeping with recommendations for defining AE surveillance and for the use of PRO-CTCAE in phase I trial design [32, 35]. Further, to ensure additional symptoms impacting tolerability are not overlooked, the option of including structured or unstructured free text reporting with characterization of severity/interference/frequency/amount attributes and symptom mapping could be considered [36]. Finally, as we and others have shown discordant patient- and physician-reporting of AEs in phase I trials, it will be essential to use the tailored PRO-CTCAE tool alongside investigator assessments of toxicities using standard CTCAE methods in phase I trial design [19, 37].

Our condensed survey addresses an unmet need within the phase I trial setting and offers several advantages in practice. It is tumor site-agnostic and can be applied to phase I trials with varying study populations and investigational agents. Our tool further leverages the benefits of the full PRO-CTCAE, which has been extensively validated.

Despite these advantages, there are limitations to our approach which should be considered. The raw data used to create descriptive statistics was obtained from our previous study of the full PRO-CTCAE in phase I trials, in which the patient population reflects the proportions of tumor types and therapies encountered at our center. A relatively limited set of investigational agents and tumour types were represented in our study population, and it is likely that this could bias the specific AEs included on our survey. Although our data is largely concordant with another description of PRO-CTCAE application in a phase I setting, validation in an independent phase I cohort might identify additional AE terms warranting inclusion [38]. At the same time, advances in cancer therapies including the creation of novel drug classes (e.g., immunotherapies, antibody-drug conjugates, radiopharmaceuticals) with unique associated AEs and modes of delivery might affect the utility of the tool and necessitate revision over time. Since our dataset included responses from patients who were surveyed repeatedly at multiple timepoints, there may further be increased representation from patients who stayed on therapy for longer and resultant impacts on our domain reliability calculations. Survey data from baseline timepoints were also included in our analysis, as we reasoned that both baseline and drug-related AEs have cumulative effects on tolerability. However, inclusion of baseline data means that responses included AEs not attributable to the investigational agent. At the same time, the relatively short time interval between survey administrations precludes assessment of cumulative or delayed toxicities impacting tolerability.

Further work will be necessary to validate our survey in practice and to ensure it is fit for the purpose of measuring tolerability prior to implementation. A prospective multicenter confirmation of the core symptom list would be valuable for ensuring reproducibility and generalizability. The analytic framework needed to translate the patients’ responses into an assessment of tolerability using a previously published composite grading algorithm will also need to be further developed [39]. Finally, and most importantly, it will be essential to capture patients’ perspectives on whether the tailored survey adequately covers AEs they feel are important, because only patients can truly define a drug’s tolerability.

## Supplementary information

Supplementary Information

## Data Availability

The data underlying this article will be shared on reasonable request to the corresponding author.
