# Supplementary material for: A tailored phase I-specific patient-reported outcome (PRO) survey to capture the patient experience of symptomatic adverse events
Source: Br J Cancer. 2023 Jul 7;129(4):612–9. doi: 10.1038/s41416-023-02307-w (PMC10421959; doi:10.1038/s41416-023-02307-w)
Supplement: Supplementary file 1 — Supplementary Information [file 41416_2023_2307_MOESM1_ESM.docx]

**SUPPLEMENTARY FIGURES**

**Supplementary Figure 1. PRO-CTCAE survey terms ranked by prevalence amongst the study population.** The dashed line denotes a threshold of 5% prevalence which was used for survey term tailoring.

**Supplementary Figure 2. PRO-CTCAE survey terms ranked by severity proportion score.** Severity proportion score was calculated by determining the proportion of patients who reported symptom severity ≥ “moderate” for a particular symptom amongst all patients who experienced that symptom. The dashed line denotes a threshold of 25% which was used for survey term tailoring. Terms in grey scale were already removed in prior rounds of survey tailoring.

**Supplementary Figure 3. PRO-CTCAE survey terms ranked by interference proportion score.** Interference proportion score was calculated by determining the proportion of patients who reported symptom interference on their usual or daily activities ≥ “somewhat” amongst all patients who experienced that symptom. The dashed line denotes a threshold of 25% which was used for survey term tailoring. Terms in grey scale were already removed in prior rounds of survey tailoring.

**Supplementary Figure 4. PRO-CTCAE survey terms ranked by frequency proportion score.** Frequency proportion score was calculated by determining the proportion of patients who reported symptom frequency ≥ “occasionally” amongst all patients who experienced that symptom. The dashed line denotes a threshold of 25% which was used for survey term tailoring. Terms in grey scale were already removed in prior rounds of survey tailoring.

**Supplementary Figure 5. PRO-CTCAE survey terms ranked by amount proportion score.** Amount proportion score was calculated by determining the proportion of patients who reported symptom amount ≥ “somewhat” amongst all patients who experienced that symptom. The dashed line denotes a threshold of 25% which was used for survey term tailoring.

**Supplementary Figure 6. PRO-CTCAE survey terms with associated percent change in domain reliability and grouped by organ system domains.** Terms in grey scale were already removed in prior rounds of survey tailoring. The single remaining survey term with lowest percent change in domain reliability from each organ system domain with $\geq2$ remaining survey terms was eliminated (identified in red shading).

**Supplementary Figure 7. Remaining PRO-CTCAE survey terms ranked by physician-reported impact proportion score.** Physician-reported impact proportion score was calculated by determining the proportion of physicians who reported symptom impact ≥ “moderate” divided by the total number of survey responses. The dashed line denotes a threshold of 25% which was used for survey term tailoring.
